# Role of Platelet-Rich Plasma in Genitourinary Syndrome of Menopause

**DOI:** 10.7759/cureus.53316

**Published:** 2024-01-31

**Authors:** Tejal Waghe, Neema Acharya, Megha Karnik, Shazia Mohammad, Nidhi A Patel, Rinkle Gemnani

**Affiliations:** 1 Department of Obstetrics and Gynecology, Jawaharlal Nehru Medical College, Datta Meghe Institute of Higher Education and Research, Wardha, IND; 2 Department of Medicine, Jawaharlal Nehru Medical College, Datta Meghe Institute of Higher Education and Research, Wardha, IND

**Keywords:** genitourinary syndrome of menopause (gsm), vascular endothelial growth factor (vegf), photodynamic therapy (pdt), hyaluronic acid (ha), platelet rich plasma (ppp)

## Abstract

The genitourinary syndrome of menopause (GSM) encompasses a range of symptoms linked to the genitourinary tract stemming from the reduction in estrogen levels following menopause. These symptoms may endure throughout a woman's lifetime. Platelet-rich plasma (PRP), known for its capacity to induce angiogenesis and the restoration effects of growth factors, has been widely employed in various disorders, including GSM. This article aims to comprehensively review the existing literature on the utilization of PRP for managing GSM. The search was executed in electronic databases, specifically PubMed, Scopus, and Google Scholar, up until April 2023. Eligible studies were meticulously chosen for inclusion in this systematic review. PRP emerges as a viable alternative for addressing vaginal atrophy, exhibiting favorable outcomes. Notably, it can be considered for patients with contraindications to hormonal therapy. However, the available body of evidence supporting the use of PRP for GSM remains limited. PRP presents itself as a promising agent, offering a patient-friendly, cost-effective alternative modality. To establish the efficacy of PRP in treating GSM definitively, future randomized trials are imperative.

## Introduction and background

Genitourinary syndrome of menopause (GSM) encompasses a range of symptoms and signs associated with decreased levels of estrogen and other sex steroids. It primarily induces changes in the labia minora/majora, vagina, vestibule, clitoris, urethra, and bladder. The genital symptoms involve dryness, burning, and irritation, while the sexual symptoms encompass a lack of lubrication and pain during intercourse. Symptoms related to the urinary tract include dysuria, urgency, and recurrent urinary tract infections. Women may present with one or more of these bothersome signs and symptoms. GSM is alternatively referred to as vulvovaginal atrophy (VVA), vaginal atrophy, and atrophic vaginitis [1]. Despite its prevalence and growing significance, GSM remains underdiagnosed and inconsistently treated. A thorough diagnostic workup is crucial for optimal therapy and follow-up.

Various treatment modalities are available for GSM, broadly categorized into hormonal and non-hormonal approaches [2]. However, the initial treatment modality of choice for GSM involves moisturizers and non-hormonal vaginal lubricants [3]. These provide immediate and temporary relief from dryness and pain during intercourse. Regular application of moisturizers enhances coital comfort and increases vaginal moisture over time [4]. In cases of moderate-to-severe GSM where conservative methods prove ineffective, hormonal therapy with estrogen products is considered the gold standard [5,6].

Hormones with local and systemic actions are available, with estriol, estradiol, prasterone, and testosterone acting locally in low doses. Caution is advised when using estrogen due to the elevated risk of thromboembolism and stroke. For breast cancer patients for whom estrogen-based therapy is contraindicated, the use of liquid lidocaine has shown promising results [7]. The risks of hormonal therapy should be thoroughly assessed in patients with gynecological cancer, considering factors such as dose, treatment modality, cancer histology, age, and prior exposure [8]. In cases of endometrial cancer, available data suggest that hormonal therapy may be considered safe in low-risk subtypes but should be avoided in high-risk subtypes [9]. Non-hormonal treatment options include moisturizers/lubricants, hyaluronic acid, colostrum gel, phytoestrogens, vaginal vitamin D/vitamin E, probiotics, laser treatment, and radiofrequency [10].

To restore tropism in the lower genitourinary tract, microablative fractional CO2 and non-ablative erbium: YAG (Er: YAG) lasers have been suggested [11-13]. Phytoestrogens, particularly equol, have shown promise in relieving menopausal symptoms, although further research is needed [14]. Prophylactic measures such as smoking cessation can be beneficial, as smoking increases estrogen metabolism, potentially leading to vaginal atrophy [15]. Recently, platelet-derived products with regenerative potential have been employed in treating GSM. Kingsley et al. first described platelet-rich plasma (PRP) in 1954 [16]. PRP, defined as plasma with platelets exceeding 1 million/μl in every 5 cc of plasma, contains growth factors and cytokines, making it valuable for tissue regeneration. The ability to induce angiogenesis and facilitate tissue repair is attributed to the supraphysiologic levels of essential growth factors released by activated platelets [17-19]. PRP was initially utilized in sports medicine and orthopedics and has expanded to various medical fields, including gynecology. The literature on the usage of PRP in patients with GSM and its safety and efficacy remains limited, necessitating further exploration. This article aims to review the existing literature on the application of PRP to managing GSM.

## Review

Methods

**Figure 1:**
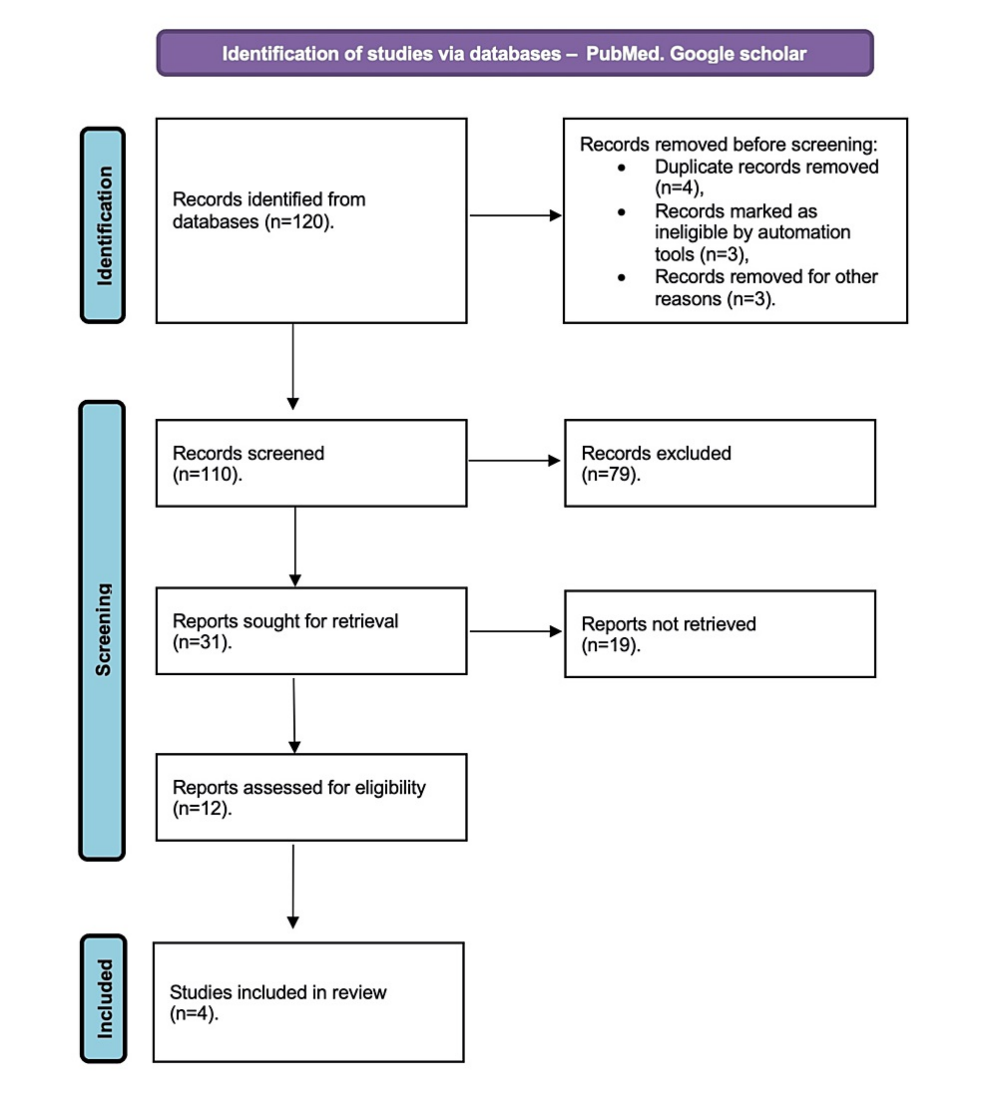
PRISMA methodology for literature search Adapted from the Preferred Reporting Items for Systematic Reviews and Meta-analysis (PRISMA) guidelines

The search for relevant articles was systematically conducted in electronic databases, including PubMed, Scopus, and Google Scholar, up to April 2023 (Figure 1). Articles specifically addressing PRP use in GSM were identified. The keywords employed in the search included "platelet-rich plasma," "PRP," "vaginal atrophy," and "GSM." Eligible studies were meticulously chosen for inclusion in this systematic review. The inclusion criteria were studies containing pertinent data on using PRP in the context of GSM. Studies were excluded if they focused on indications for PRP use other than GSM, lacked a clear description of the administration method, had a brief follow-up period, or failed to present their results adequately. Adhering to the Preferred Reporting Items for Systematic Reviews and Meta-Analyses (PRISMA) guidelines, this review followed a structured and transparent methodology.

Review

In a case report by Kim et al. (2017), a 67-year-old female experiencing vaginal itching, irritation for five years, and poor cosmetic appearance of her external genitalia initially attempted estrogen treatment with no symptomatic relief. A SmartPreP® APC-30 kit prepared 4 ml of autologous PRP from 30 ml of whole blood and injected it into the labia majora subcutaneous layer. Within a month, pruritus and irritation disappeared, and there was noticeable volume augmentation of the labia majora in the immediate postoperative period [20].

A prospective phase II pilot study conducted by Hersant et al. (2018) included twenty breast cancer survivors affected by VVA. These patients, with a Gloria Bachman vaginal health index (VHI) score of <15, received an A-PRP+HA (Regenkit) combination intramucosally. Clinical evaluations were performed at 0, 1, 3, and 6 months, utilizing VHI and female sexual distress (FSD) scores. Improvement in clinical symptoms of vaginal dryness and dyspareunia was observed, with a significant increase in VHI scores at six months (p<0.0001) and a notable decrease in FSD scores during the study (p<0.0001) [21].

A randomized control trial by Surkichin et al. (2021) assessed the efficacy of PDT in combination with PRP for GSM. Fifteen patients received 10 sessions of photosensitizer chlorin E6 gel and physiotherapy, improving itching, dryness, and dyspareunia. Significant enhancements were noted in VHI, visual analog scale (VAS), and dermatology life quality index (DLQI), indicating improved vaginal health, epithelial integrity, moisture, transudate, pH, and visual indicators of vulvar area changes [22].

A study conducted by Saleh et al. (2022) demonstrated a substantial enhancement in postmenopausal VVA through autologous PRP (A-PRP) injection. This improvement was evident in the significant enhancement of both the total VHI score and its components one month after treatment (p<0.001). Additionally, there was notable progress in specific aspects such as burning, pain, irritation, dryness, discharge, desire for intimacy, sexual relationships, pain during sexual activity, and dryness during sexual activity at the one-month mark post-treatment, as revealed by the VSQ (p=0.045 for dryness and p<0.001 for other items) [23].

A pilot study conducted by Long et al. (2021) investigated the effectiveness of A-PR) injections as a treatment for women with stress urinary incontinence (SUI). Twenty women with SUI received A-PRP injections at the anterior vaginal wall near the mid-urethra. Symptom severity was evaluated using self-reported questionnaires before treatment and at one and six months post-treatment. The study found significant improvement in incontinence symptoms at both time points, with no significant adverse reactions. While younger women seemed to have slightly better outcomes, this observation did not reach statistical significance. The treatment showed no significant effect on sexual function. This research suggests that A-PRP injections could be a safe and somewhat effective treatment for mild to moderate SUI in women, opening avenues for further exploration in this field [24].

A systematic review was done by Prodromidou et al. (2021), which focuses on evaluating the effectiveness of PRP in treating urogynecological disorders. A comprehensive literature review was conducted to assess PRP's role in managing conditions like vaginal atrophy, pelvic organ prolapse, urinary incontinence, vaginal fistulas, and mesh exposure. The results show PRP as a feasible alternative, especially in cases where hormone therapy is contraindicated. However, the study concludes that more extensive randomized trials are necessary to fully establish PRP's efficacy in these treatments [25].

Vaginal atrophy, often resulting from aging and perineal trauma, significantly affects women's health. A review conducted by Zheng et al. (2021) examines a novel treatment approach involving the injection of various materials into the vaginal wall, including PRP, autogenous fat graft, hyaluronic acid, botulinum toxin, and collagen. These materials show potential for restoring vaginal function, augmenting the vaginal wall, and reducing pain during sexual intercourse. Preliminary results indicate this technique's effectiveness and safety, primarily in mild cases of vaginal atrophy or as an adjunct to surgery. However, the risk of vascular embolisms as a serious complication and the lack of extensive studies, particularly randomized double-blind placebo-controlled trials, necessitate further research to establish definitive efficacy and safety profiles and to standardize treatment protocols [26].

Table 1 shows the summary of the characteristics of the studies for the use of PRP in GSM.

**Table 1:** Characteristics of the studies for the use of PRP in GSM PRP: platelet-rich plasma, VVA: vulvovaginal atrophy, PDT: photodynamic therapy, HA: hyaluronic acid, VSQ: vulvovaginal symptom questionnaire, GSM: genitourinary syndrome of menopause

Title of article	Author(s)	Year	Technique	Result	Conclusion
Rejuvenation using platelet-rich plasma and Lipofilling for vaginal atrophy and lichen sclerosis [20]	Kim et al.	2017	The subcutaneous layer of the labia majora is aseptically injected at four sites.	Relief of symptoms, satisfaction with the change in contour, and youthful appearance of the external genitalia. Application of autologous lipofilling with PRP led to symptom relief, resolution of lichen sclerosus on the labia minora, and restoration of labia majora contour.	The combination of autologous lipofilling with PRP is proposed as an effective modality for treating vaginal atrophy and lichen sclerosus.
Efficacy of injecting platelet concentrate combined with hyaluronic acid for the treatment of vulvovaginal atrophy in postmenopausal women with a history of breast cancer: a phase 2 pilot study [21]	Hersant et al.	2018	4 mL of PRP-HA was injected into the vestibule and the first 3 cm of the vagina using a point-by-point technique (0.1 mL/point).	Improvement in clinical symptoms of vaginal dryness and dyspareunia.	The injection of A-PRP-HA appears to be a promising method for enhancing trophicity and hydration of vaginal mucosa in the treatment of VVA in postmenopausal breast cancer survivors with contraindications to hormone therapy.
Evaluation of the effectiveness of the combined use of photodynamic therapy and platelet-rich plasma in the genitourinary syndrome of menopause [22]	Surkichin et al.	2021	The PDT Harmony device was utilized as a source of radiation.	Significant reduction of main complaints such as dryness and itching, and improved visual indicators of changes in the vulvar area.	The combined use of PDT and PRP therapy demonstrated high efficiency in significantly reducing major complaints like dryness and itching and improving visual indicators of vulvar area changes in GSM.
Clinical evaluation of autologous platelet-rich plasma injection in postmenopausal vulvovaginal atrophy: a pilot study [23]	Saleh et al.	2022	47 women with postmenopausal VVA underwent two sessions of A-PRP injections, evaluated using the VHI and VSQ.	Significant improvements in VVA symptoms were observed post-treatment, with notable enhancements in VHI scores and VSQ items, including sexual function and quality of life.	A-PRP injections are effective and safe for treating postmenopausal VVA, offering a viable hormone-free option for vulvovaginal rejuvenation.
A pilot study: effectiveness of local injection of autologous platelet‑rich plasma in treating women with stress urinary incontinence [24]	Long et al.	2021	The technique involved injecting A-PRP into the anterior vaginal wall near the mid-urethra.	Significant improvement in incontinence at 1 and 6 months post-treatment, assessed by ICIQ-SF, UDI-6, IIQ-7, and OABSS. No change in POPDI-6 scores​​.	A-PRP injection is safe and somewhat effective for female SUI at 1 and 6 months. Potential better outcomes were seen in younger women.
The emerging role on the use of platelet-rich plasma products in the management of urogynaecological disorders [25]	Prodromidou et al.	2021	It is a systematic review using a meticulous literature search using three electronic databases to synthesize evidence of PRP products in managing urogynaecology disorders.	PRP showed improvement in vaginal atrophy, collagen increase in pelvic organ prolapse, symptom relief in urinary incontinence, and benefits in treating vaginal fistulas and mesh extrusion.	PRP is a promising, easy-to-apply, cost-effective, and feasible alternative therapeutic modality for various urogynaecology disorders, but further large-scale studies are needed for definitive evidence.
Materials selection for the injection into vaginal wall for treatment of vaginal atrophy [26]	Zheng et al.	2021	It is a systematic review involving the injection of various materials into the vaginal wall as a treatment for vaginal atrophy. The materials used in this technique include PRP, autogenous fat graft, HA, botulinum toxin, and collagen.	PRP: requires multiple injections but has shown promising results in restoring vaginal function. HA, autogenous fat graft, and collagen: mainly effective in augmenting the vaginal wall, enhancing its structure and resilience. Botulinum toxin: provides significant relief from pain during sexual intercourse by reducing vaginal muscle spasms, especially beneficial for patients with vaginismus.	The innovative technique of injecting materials like PRP, autogenous fat graft, HA, botulinum toxin, and collagen into the vaginal wall shows promise in treating vaginal atrophy, especially in milder cases or as an adjunct to surgery. It needs further studies to establish efficacy, safety, and standardized procedures for the benefit of a wider patient population.

Future directions

Future research endeavors should prioritize comprehensive randomized trials to elucidate PRP's efficacy and potential nuances in treating the GSM. These trials should explore the standalone application of PRP and delve into its comparative effectiveness when juxtaposed with other therapeutic modalities. Moreover, efforts should be directed toward establishing standardized protocols for PRP administration and assessing optimal dosages and frequencies to maximize therapeutic benefits while ensuring patient safety. Long-term follow-up studies are essential to gauge the observed positive outcomes' sustainability and monitor potential side effects or complications. In addition, investigating the underlying mechanisms of action of PRP in conjunction with other modalities will contribute to a deeper understanding of the biological processes involved in ameliorating symptoms associated with the GSM. Collaborative interdisciplinary studies involving gynecologists, urologists, oncologists, and researchers in regenerative medicine will foster a holistic approach to refining treatment strategies. Ultimately, these future directions aim to solidify the role of PRP as a reliable and effective therapeutic option, offering valuable insights for clinical practitioners and enhancing the overall quality of care for individuals experiencing the GSM.

Limitation

However insightful, the studies reviewed in this article reveal certain limitations that warrant consideration. The predominant focus on the combined application of PRP with other therapeutic modalities leaves a notable gap in evidence regarding the standalone efficacy of PRP in treating the GSM. Moreover, the heterogeneity in study designs, encompassing variations in sample sizes and methodologies, introduces potential biases and challenges in drawing universally applicable conclusions. The absence of comprehensive long-term follow-up data across several studies hinders the assessment of the sustainability of treatment effects and the identification of any delayed complications. Additionally, the diversity observed in patient populations, including variations in age, medical history, and symptom severity, raises concerns about the generalizability of findings to a broader demographic. Finally, the potential influence of publication bias, where positive results are more likely to be published, poses a risk to the overall objectivity of the assessment. Addressing these limitations through standardized study designs, larger and more homogeneous sample sizes, and extended follow-up periods will be instrumental in advancing the understanding of both the benefits and limitations of PRP as a therapeutic option for the GSM.

## Conclusions

The amalgamation of PRP with various modalities, including autologous lipofilling, hyaluronic acid, and photodynamic therapy, has demonstrated promising results in treating the genitourinary syndrome of menopause. Positive outcomes were observed, such as improved symptoms associated with vaginal atrophy, contour restoration on the labia majora when combined with autologous lipofilling, and enhanced trophicity and hydration of the vaginal mucosa in postmenopausal breast cancer survivor patients when used with hyaluronic acid in females with SUI and urogynecological disorders. While significant regression of symptoms like itching and dryness was noted with the combination of PRP and photodynamic therapy, it is crucial to acknowledge that limited evidence exists for the standalone use of PRP in treating GSM. Consequently, the necessity for future randomized trials is emphasized to affirm the effectiveness of PRP as a promising, practical, and cost-effective modality in managing this syndrome.
